# 7-Chloro-1,2-dihydro­furo[2,3-*c*]isoquinolin-5-amine

**DOI:** 10.1107/S160053681004273X

**Published:** 2010-10-30

**Authors:** Kensuke Okuda, Takashi Hirota, Kenji Sasaki, Hiroyuki Ishida

**Affiliations:** a Laboratory of Medicinal and Pharmaceutical Chemistry, Gifu Pharmaceutical University, Gifu 501-1196, Japan; b Faculty of Pharmaceutical Sciences, Okayama University, Okayama 700-8530, Japan; c Department of Chemistry, Faculty of Science, Okayama University, Okayama 700-8530, Japan

## Abstract

In the title compound, C_11_H_9_ClN_2_O, the fused-ring system is essentially planar, with a maximum deviation of 0.0323 (16) Å. In the crystal, mol­ecules are connected by N—H⋯O hydrogen bonds forming a zigzag chain along the *c* axis. Mol­ecules are further stacked along the *a* axis through weak π–π inter­actions, the shortest distance between ring centroids being 3.6476 (8) Å.

## Related literature

For background to this work and the synthesis of the title compound, see: Okuda *et al.* (2010 ▶).
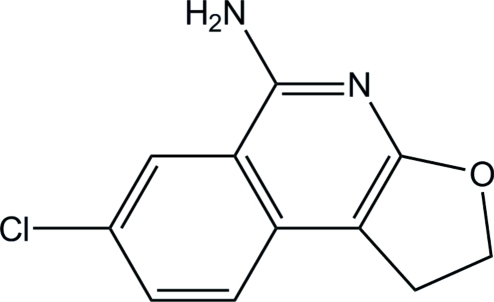


## Experimental

### 

#### Crystal data

C_11_H_9_ClN_2_O
*M* *_r_* = 220.66
Orthorhombic, 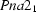

*a* = 7.2948 (6) Å
*b* = 12.0703 (11) Å
*c* = 10.8869 (8) Å
*V* = 958.60 (14) Å^3^
*Z* = 4
Mo *K*α radiation
μ = 0.37 mm^−1^
*T* = 200 K
0.40 × 0.25 × 0.08 mm

#### Data collection

Rigaku R-AXIS RAPID II diffractometer
Absorption correction: numerical (*NUMABS*; Higashi, 1999 ▶) *T*_min_ = 0.896, *T*_max_ = 0.971
11594 measured reflections
2776 independent reflections
2531 reflections with *I* > 2σ(*I*)
*R*_int_ = 0.016

#### Refinement

*R*[*F* ^2^ > 2σ(*F* ^2^)] = 0.031
*wR*(*F* ^2^) = 0.083
*S* = 1.11
2776 reflections
144 parameters
1 restraint
H atoms treated by a mixture of independent and constrained refinement
Δρ_max_ = 0.42 e Å^−3^
Δρ_min_ = −0.17 e Å^−3^
Absolute structure: Flack (1983 ▶), 1311 Friedel pairs
Flack parameter: −0.02 (5)

### 

Data collection: *PROCESS-AUTO* (Rigaku/MSC, 2004 ▶); cell refinement: *PROCESS-AUTO*; data reduction: *CrystalStructure* (Rigaku/MSC, 2004 ▶); program(s) used to solve structure: *SHELXS97* (Sheldrick, 2008 ▶); program(s) used to refine structure: *SHELXL97* (Sheldrick, 2008 ▶); molecular graphics: *ORTEP-3* (Farrugia, 1997 ▶); software used to prepare material for publication: *CrystalStructure* (Rigaku/MSC, 2004 ▶) and *PLATON* (Spek, 2009 ▶).

## Supplementary Material

Crystal structure: contains datablocks global, I. DOI: 10.1107/S160053681004273X/gk2312sup1.cif

Structure factors: contains datablocks I. DOI: 10.1107/S160053681004273X/gk2312Isup2.hkl

Additional supplementary materials:  crystallographic information; 3D view; checkCIF report

## Figures and Tables

**Table 1:** Hydrogen-bond geometry (Å, °)

*D*—H⋯*A*	*D*—H	H⋯*A*	*D*⋯*A*	*D*—H⋯*A*
N2—H2*A*⋯O1^i^	0.82 (2)	2.43 (2)	3.062 (2)	134 (2)

Symmetry code: (i) 
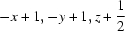
.

**Fig. 1.**
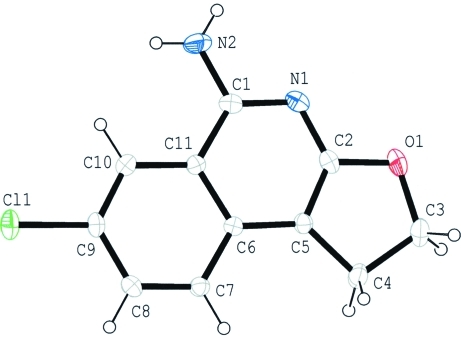
The molecular structure of the title compound, with the atom-labeling. Displacement ellipsoids of non-H atoms are drawn at the 50% probability level.

**Fig. 2.**
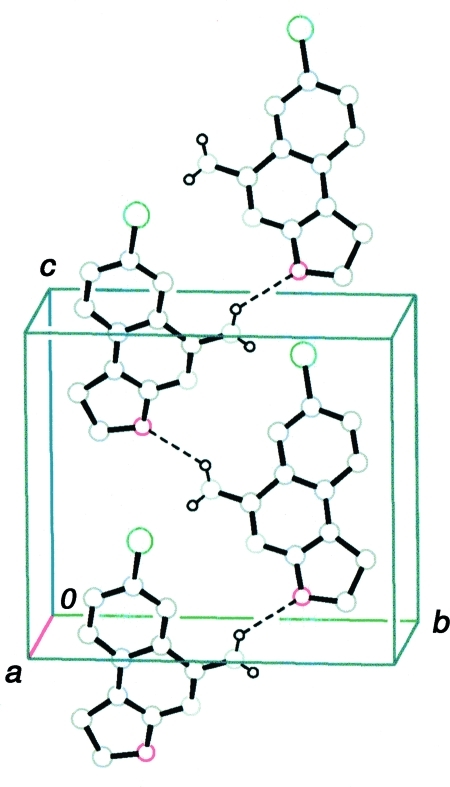
A packing diagram of the title compound, showing a molecular chain running along the c axis. The dashed lines indicate N—H···O hydrogen bonds. C-bound H atoms have been omitted.

## supplementary crystallographic information

### Comment

As an extension of our work to develop complex heterocyclic skeletons for potential pharmaceutics in one step using Truce-Smiles rearrangement, we got interested in the reaction of 2-(3-cyanopropoxy)benzonitriles with bases (Okuda *et al.*, 2010). As is well established, the key step of the Truce-Smiles rearrangement is the *ipso* attack of an incoming nucleophile. So electron withdrawing chlorine atom at 5-position seemed to be favorable for this rearrangement reaction. The product, 7-chloro-1,2-dihydrofuro[2,3-*c*]isoquinolin-5-amine, was obtained in 83% yield, which was higher than the product yield of 1,2-dihydrofuro[2,3-*c*]isoquinolin-5-amine (60%) from 5-unsubsituted starting material as we had assumed.

In the title compound, C_11_H_9_ClN_2_O, the fused three-ring system is essentially planar with a maximum deviation of 0.0323 (16) Å at atom C4. In the crystal structure, the molecules are connected by an N—H···O hydrogen bond, forming a zigzag chain along the *c* axis. The molecules are further stacked along the *a* axis through weak π–π interactions between the isoquinoline ring systems [*Cg*1···*Cg*1 (-1/2 + *x*, 3/2 - *y*, *z*) = 4.0137 (8) Å, *Cg*1···*Cg*2 (1/2 + *x*, 3/2 - *y*, *z*) = 3.6858 (8) Å and *Cg*2···*Cg*2 (1/2 + *x*, 3/2 - *y*, *z*) = 3.6476 (8) Å; *Cg*1 and *Cg*2 are the centroids of N1/C1/C11/C6/C5/C2 and C6–C11 rings, respectively.]

### Experimental

Detailed experimental procedure of the synthesis of 7-chloro-1,2-dihydrofuro[2,3-*c*]isoquinolin-5-amine (m.p. 527–528 K from ethyl acetate) from 5-chloro-2-(3-cyanopropoxy)benzonitrile was already described in our precedent report (Okuda *et al.*, 2010). Single crystals suitable for X-ray diffraction were obtained from an ethyl acetate solution.

### Refinement

C-bound H atoms were positioned geometrically (C—H = 0.95 or 0.99 Å) and refined as riding, with *U*_iso_(H) = 1.2*U*_eq_(C). The N-bound H atoms were found in a difference Fourier map and refined isotropically. The refined N—H distancea are 0.80 (3) anf 0.82 (3) Å. The Hooft *y* parameter value is -0.002 (13).

### Crystal data

**Table:**

C_11_H_9_ClN_2_O	*F*(000) = 456.00
*M**_r_* = 220.66	*D*_x_ = 1.529 Mg m^−^^3^
Orthorhombic, *P**n**a*2_1_	Mo *K*α radiation, λ = 0.71075 Å
Hall symbol: P 2c -2n	Cell parameters from 10138 reflections
*a* = 7.2948 (6) Å	θ = 3.3–30.0°
*b* = 12.0703 (11) Å	µ = 0.37 mm^−^^1^
*c* = 10.8869 (8) Å	*T* = 200 K
*V* = 958.60 (14) Å^3^	Platelet, yellow
*Z* = 4	0.40 × 0.25 × 0.08 mm

### Data collection

**Table:**

Rigaku R-AXIS RAPID II diffractometer	2531 reflections with *I* > 2σ(*I*)
Detector resolution: 10.00 pixels mm^-1^	*R*_int_ = 0.016
ω scans	θ_max_ = 30.0°
Absorption correction: numerical (*NUMABS*; Higashi, 1999)	*h* = −10→9
*T*_min_ = 0.896, *T*_max_ = 0.971	*k* = −16→16
11594 measured reflections	*l* = −15→15
2776 independent reflections	

### Refinement

**Table:**

Refinement on *F*^2^	Secondary atom site location: difference Fourier map
Least-squares matrix: full	Hydrogen site location: inferred from neighbouring sites
*R*[*F*^2^ > 2σ(*F*^2^)] = 0.031	H atoms treated by a mixture of independent and constrained refinement
*wR*(*F*^2^) = 0.083	*w* = 1/[σ^2^(*F*_o_^2^) + (0.056*P*)^2^ + 0.0168*P*] where *P* = (*F*_o_^2^ + 2*F*_c_^2^)/3
*S* = 1.11	(Δ/σ)_max_ = 0.001
2776 reflections	Δρ_max_ = 0.42 e Å^−^^3^
144 parameters	Δρ_min_ = −0.17 e Å^−^^3^
1 restraint	Absolute structure: Flack (1983), 1311 Friedel pairs
Primary atom site location: structure-invariant direct methods	Flack parameter: −0.02 (5)

### Special details

**Table:**

Geometry. All e.s.d.'s (except the e.s.d. in the dihedral angle between two l.s. planes) are estimated using the full covariance matrix. The cell e.s.d.'s are taken into account individually in the estimation of e.s.d.'s in distances, angles and torsion angles; correlations between e.s.d.'s in cell parameters are only used when they are defined by crystal symmetry. An approximate (isotropic) treatment of cell e.s.d.'s is used for estimating e.s.d.'s involving l.s. planes.
Refinement. Refinement of *F*^2^ against ALL reflections. The weighted *R*-factor *wR* and goodness of fit *S* are based on *F*^2^, conventional *R*-factors *R* are based on *F*, with *F* set to zero for negative *F*^2^. The threshold expression of *F*^2^ > σ(*F*^2^) is used only for calculating *R*-factors(gt) *etc*. and is not relevant to the choice of reflections for refinement. *R*-factors based on *F*^2^ are statistically about twice as large as those based on *F*, and *R*- factors based on ALL data will be even larger.

### Fractional atomic coordinates and isotropic or equivalent isotropic displacement parameters (Å^2^)

**Table:**

	*x*	*y*	*z*	*U*_iso_*/*U*_eq_	
Cl1	0.20651 (5)	0.70539 (3)	0.83910 (3)	0.04205 (11)	
O1	0.5523 (2)	0.71804 (10)	0.14408 (11)	0.0454 (3)	
N1	0.49290 (17)	0.58990 (10)	0.29743 (12)	0.0364 (3)	
N2	0.4320 (3)	0.46639 (12)	0.4512 (2)	0.0522 (4)	
C1	0.44057 (18)	0.57365 (11)	0.41337 (13)	0.0328 (3)	
C2	0.49833 (18)	0.69629 (12)	0.26182 (13)	0.0317 (3)	
C3	0.5446 (2)	0.83764 (15)	0.12840 (14)	0.0431 (3)	
H3A	0.4551	0.8569	0.0635	0.052*	
H3B	0.6664	0.8662	0.1037	0.052*	
C4	0.4862 (2)	0.88976 (13)	0.25161 (14)	0.0375 (3)	
H4A	0.5839	0.9376	0.2858	0.045*	
H4B	0.3720	0.9333	0.2430	0.045*	
C5	0.45715 (16)	0.78864 (9)	0.32871 (13)	0.0267 (2)	
C6	0.39999 (16)	0.77295 (10)	0.45094 (12)	0.0239 (2)	
C7	0.35257 (17)	0.86096 (10)	0.53113 (12)	0.0279 (2)	
H7	0.3619	0.9353	0.5030	0.033*	
C8	0.29337 (17)	0.84059 (12)	0.64864 (12)	0.0302 (3)	
H8	0.2602	0.9000	0.7013	0.036*	
C9	0.28264 (17)	0.73061 (12)	0.68982 (13)	0.0297 (3)	
C10	0.32916 (18)	0.64268 (11)	0.61666 (12)	0.0297 (3)	
H10	0.3215	0.5692	0.6474	0.036*	
C11	0.38839 (17)	0.66225 (10)	0.49559 (12)	0.0258 (2)	
H2A	0.430 (3)	0.4520 (19)	0.525 (2)	0.054 (6)*	
H2B	0.466 (4)	0.418 (2)	0.406 (2)	0.069 (8)*	

### Atomic displacement parameters (Å^2^)

**Table:**

	*U*^11^	*U*^22^	*U*^33^	*U*^12^	*U*^13^	*U*^23^
Cl1	0.04108 (18)	0.0588 (2)	0.02626 (15)	0.00458 (13)	0.00713 (15)	0.00747 (16)
O1	0.0576 (7)	0.0536 (7)	0.0249 (5)	0.0026 (5)	0.0091 (5)	−0.0055 (4)
N1	0.0402 (6)	0.0329 (6)	0.0361 (6)	0.0028 (4)	0.0018 (5)	−0.0093 (5)
N2	0.0730 (10)	0.0248 (6)	0.0588 (10)	0.0045 (6)	0.0114 (8)	−0.0006 (6)
C1	0.0332 (6)	0.0269 (6)	0.0382 (7)	0.0012 (5)	−0.0001 (5)	−0.0036 (5)
C2	0.0300 (6)	0.0396 (7)	0.0254 (6)	0.0021 (5)	0.0013 (5)	−0.0046 (5)
C3	0.0492 (8)	0.0533 (9)	0.0267 (6)	0.0020 (7)	0.0044 (6)	0.0073 (6)
C4	0.0435 (7)	0.0388 (7)	0.0301 (6)	0.0003 (6)	0.0057 (6)	0.0078 (5)
C5	0.0249 (5)	0.0298 (5)	0.0254 (6)	0.0010 (4)	0.0018 (5)	0.0003 (5)
C6	0.0208 (5)	0.0251 (5)	0.0257 (5)	0.0002 (4)	−0.0004 (4)	−0.0004 (4)
C7	0.0291 (5)	0.0253 (6)	0.0292 (6)	0.0010 (4)	0.0021 (5)	0.0003 (4)
C8	0.0287 (6)	0.0335 (6)	0.0285 (6)	0.0018 (4)	0.0013 (5)	−0.0058 (5)
C9	0.0244 (6)	0.0420 (7)	0.0227 (5)	0.0000 (5)	0.0015 (4)	0.0028 (5)
C10	0.0287 (5)	0.0292 (6)	0.0312 (6)	0.0002 (4)	0.0005 (5)	0.0046 (5)
C11	0.0247 (5)	0.0260 (5)	0.0268 (5)	0.0001 (4)	−0.0016 (4)	0.0006 (5)

### Geometric parameters (Å, °)

**Table:**

Cl1—C9	1.7442 (14)	C4—C5	1.4964 (19)
O1—C2	1.3664 (19)	C4—H4A	0.9900
O1—C3	1.455 (2)	C4—H4B	0.9900
N1—C1	1.3332 (19)	C5—C6	1.4074 (19)
N1—C2	1.3420 (19)	C6—C7	1.4178 (16)
N2—C1	1.3599 (19)	C6—C11	1.4244 (17)
N2—H2A	0.82 (3)	C7—C8	1.3725 (19)
N2—H2B	0.80 (3)	C7—H7	0.9500
C1—C11	1.4457 (18)	C8—C9	1.403 (2)
C2—C5	1.3649 (18)	C8—H8	0.9500
C3—C4	1.542 (2)	C9—C10	1.370 (2)
C3—H3A	0.9900	C10—C11	1.4070 (18)
C3—H3B	0.9900	C10—H10	0.9500
			
C2—O1—C3	106.83 (11)	H4A—C4—H4B	109.3
C1—N1—C2	115.01 (12)	C2—C5—C6	117.37 (12)
C1—N2—H2A	119.8 (17)	C2—C5—C4	109.61 (13)
C1—N2—H2B	119.1 (18)	C6—C5—C4	133.01 (12)
H2A—N2—H2B	117 (2)	C5—C6—C7	123.62 (11)
N1—C1—N2	116.10 (14)	C5—C6—C11	117.81 (11)
N1—C1—C11	123.56 (12)	C7—C6—C11	118.57 (11)
N2—C1—C11	120.31 (14)	C8—C7—C6	121.09 (11)
N1—C2—C5	128.38 (14)	C8—C7—H7	119.5
N1—C2—O1	117.60 (12)	C6—C7—H7	119.5
C5—C2—O1	114.02 (13)	C7—C8—C9	119.00 (12)
O1—C3—C4	108.27 (11)	C7—C8—H8	120.5
O1—C3—H3A	110.0	C9—C8—H8	120.5
C4—C3—H3A	110.0	C10—C9—C8	122.23 (13)
O1—C3—H3B	110.0	C10—C9—Cl1	119.05 (11)
C4—C3—H3B	110.0	C8—C9—Cl1	118.72 (11)
H3A—C3—H3B	108.4	C9—C10—C11	119.39 (12)
C5—C4—C3	101.21 (12)	C9—C10—H10	120.3
C5—C4—H4A	111.5	C11—C10—H10	120.3
C3—C4—H4A	111.5	C10—C11—C6	119.69 (11)
C5—C4—H4B	111.5	C10—C11—C1	122.46 (11)
C3—C4—H4B	111.5	C6—C11—C1	117.84 (12)
			
C2—N1—C1—N2	179.28 (16)	C5—C6—C7—C8	178.39 (12)
C2—N1—C1—C11	1.50 (19)	C11—C6—C7—C8	−1.19 (18)
C1—N1—C2—C5	−0.1 (2)	C6—C7—C8—C9	0.94 (19)
C1—N1—C2—O1	179.54 (13)	C7—C8—C9—C10	0.0 (2)
C3—O1—C2—N1	179.39 (14)	C7—C8—C9—Cl1	−179.77 (10)
C3—O1—C2—C5	−0.90 (17)	C8—C9—C10—C11	−0.57 (19)
C2—O1—C3—C4	2.07 (17)	Cl1—C9—C10—C11	179.17 (10)
O1—C3—C4—C5	−2.34 (17)	C9—C10—C11—C6	0.29 (18)
N1—C2—C5—C6	−0.5 (2)	C9—C10—C11—C1	179.68 (12)
O1—C2—C5—C6	179.87 (11)	C5—C6—C11—C10	−179.03 (11)
N1—C2—C5—C4	178.97 (15)	C7—C6—C11—C10	0.57 (17)
O1—C2—C5—C4	−0.69 (17)	C5—C6—C11—C1	1.54 (16)
C3—C4—C5—C2	1.84 (16)	C7—C6—C11—C1	−178.85 (11)
C3—C4—C5—C6	−178.84 (13)	N1—C1—C11—C10	178.34 (13)
C2—C5—C6—C7	−179.90 (12)	N2—C1—C11—C10	0.6 (2)
C4—C5—C6—C7	0.8 (2)	N1—C1—C11—C6	−2.26 (18)
C2—C5—C6—C11	−0.32 (17)	N2—C1—C11—C6	−179.95 (15)
C4—C5—C6—C11	−179.60 (14)		

### Hydrogen-bond geometry (Å, °)

**Table:**

*D*—H···*A*	*D*—H	H···*A*	*D*···*A*	*D*—H···*A*
N2—H2A···O1^i^	0.82 (2)	2.43 (2)	3.062 (2)	134 (2)

Symmetry codes: (i) −*x*+1, −*y*+1, *z*+1/2.
